# Pachychoroid-Related Pigment Epithelial Detachment Treated with Photodynamic Therapy

**DOI:** 10.3390/biomedicines14030620

**Published:** 2026-03-10

**Authors:** Maciej Gawęcki, Karolina Mach, Krzysztof Kiciński, Andrzej Grzybowski

**Affiliations:** 1 Dobry Wzrok Ophthalmological Center, 80-392 Gdansk, Poland; karolina.mach@dobry-wzrok.pl (K.M.); krzysztofkg999@icloud.com (K.K.); 2 Department of Ophthalmology, Pomeranian Hospitals in Wejherowo, 84-100 Wejherowo, Poland; 3 Department of Ophthalmology, University of Warmia and Mazury, Oczapowskiego 2, 10-719 Olsztyn, Poland; ae.grzybowski@gmail.com; 4 Institute for Research in Ophthalmology, Foundation for Ophthalmology Development, Mickiewicza 24, 61-836 Poznan, Poland

**Keywords:** pachychoroid, pigment epithelial detachment, pachychoroid pigment epitheliopathy, photodynamic therapy

## Abstract

**Background:** Pachychoroid pigment epitheliopathy (PPE) is a non-exudative entity within the pachychoroid disease spectrum characterized by increased choroidal thickness and isolated serous pigment epithelial detachment (PED) without subretinal fluid. Although photodynamic therapy (PDT) is established for chronic central serous chorioretinopathy (CSC), its efficacy in isolated pachychoroid-related PED remains insufficiently defined, with available evidence limited to small case series. **Purpose:** This study aims to characterize symptomatic pachychoroid-related PED and evaluate anatomical and functional outcomes following half-dose PDT (hd-PDT), with additional analysis according to lesion localization and CSC history. **Methods:** This retrospective study included 34 eyes of 27 patients treated with hd-PDT between June 2022 and December 2024. PEDs were categorized as central (fovea-involving) or paramacular. Best-corrected visual acuity (BCVA) and spectral-domain optical coherence tomography parameters—central subfield thickness (CST), mean subfield thickness (MST), macular volume (MV), subfoveal choroidal thickness (SFCT), and PED height—were assessed at baseline, 1 month, and 6 months. Treatment planning was based on indocyanine green angiography (ICGA) and spectral-domain optical coherence tomography (SD-OCT) findings. Statistical analyses employed non-parametric tests and generalized estimating equations. **Results:** Central lesions were associated with longer disease duration, worse baseline BCVA, and greater retinal thickness and PED height (*p* < 0.05). Complete PED resorption occurred in 79.4% of eyes at 1 month and 73.5% at 6 months (central: 86.3% and 81.8%; paramacular: 66.6% and 58.3%). Mean BCVA improved significantly from 0.22 ± 0.24 to 0.10 ± 0.16 logMAR at 6 months (*p* < 0.0001), with greater functional gain in central lesions. Significant reductions were observed in CST, MST, MV, and PED height, whereas SFCT remained stable. Better final BCVA correlated with younger age, shorter disease duration, smaller baseline retinal volume, smaller PDT spot size, and absence of CSC history. Non-responders had worse baseline BCVA, higher PED height, and larger treatment areas. No treatment-related complications were detected. **Conclusions:** Half-dose PDT was associated with favorable anatomical and functional outcomes in symptomatic pachychoroid-related PED, particularly in centrally located lesions. Baseline disease severity appeared to influence treatment response. Prospective studies with longer follow-up are warranted to confirm long-term efficacy and safety.

## 1. Introduction

Sole pigment epithelial detachment (PED) related to increased choroidal thickness belongs to the subtype of pachychoroid-related disorders termed pachychoroid pigment epitheliopathy (PPE). This clinical entity was first clearly described by Warrow et al. in 2013 [1]. Since then, PPE has been recognized in all classifications of pachychoroid spectrum diseases [2,3,4]. PPE is characterized by the absence of subretinal fluid (SRF) and the presence of retinal pigment epithelium (RPE) alterations such as mottling, clumping, and serous PEDs [5]. Isolated PED recognized as PPE can be accompanied by other pigmentary abnormalities; however, it can also occur as a lone symptom. Although isolated PED is considered a variant of PPE, it is also recognized as a symptom occurring during the course of central serous chorioretinopathy (CSC) [4,6]. Current recommendations for PPE treatment are not explicit. Hence, the management of PPE is usually based on recommendations for treating CSC, especially cases presenting with accompanying PED. Recent publications advise the use of photodynamic therapy (PDT) in symptomatic chronic cases, while observation is recommended for short-lasting forms of CSC or in therapy-resistant eyes without significant subjective symptoms or disease progression [4,7,8,9]. So far, just a few papers dedicated specifically to the management of isolated PED phenomenon have been published [Gupta 2013, Arif et al. 2018, Goto et al. 2012, Hwang et al. 2019, Kishimoto 2025, Tang et al. 2022] [6,10,11,12,13,14].

This study aimed to characterize symptomatic pachychoroid-related PED and to evaluate the efficacy of half-dose photodynamic therapy (hd-PDT) in this clinical entity. Additionally, we examined treatment outcomes in relation to PED location and prior history of CSC, with the aim of identifying clinical factors potentially predictive of therapeutic response.

## 2. Material and Methods

The study was approved by the local bioethical commission of Okręgowa Izba Lekarska (approval number KB-35/2023, dated 16 August 2023). All procedures in the study were performed in accordance with the Declaration of Helsinki.

The retrospective analysis included all consecutive symptomatic cases of serous PED associated with pachychoroid who consented to PDT between June 2022 and December of 2024. All patients were treated in a single ophthalmological center (Dobry Wzrok, Gdansk, Poland).

The study material comprised 34 eyes of 27 patients who were diagnosed with PPE and treated with PDT between June 2022 and December 2024. Eyes were divided into two groups according to location of the PED: central (involving the fovea) and paramacular (outside the fovea). In this cohort only one PED lesion per eye was detected. Baseline characteristics of the whole study group are provided in Table 1.

Diagnostic criteria adopted for recognition of PPE were as follows: serous PED without SRF, increased subfoveal choroidal thickness >350 μm, pigment abnormalities detected on fundus autofluorescence (FAF), increased hyperpermeability of choriocapillaris on indocyanine green angiography (ICGA), choroidal vessel dilation on spectral-domain optical coherence tomography (SD-OCT), and lack of macular neovascularization (MNV). Patients with PED were divided into subfoveal and paramacular groups depending on the location of PED.

Diagnostic tests performed at presentation included best-corrected visual acuity (BCVA) testing on a logMAR chart, SD-OCT and OCT-angiography (OCTA) (REVO FC 130 system (2023, Optopol Technology, Zawiercie, Poland)), color fundus photography (CFP), FAF, fluorescein angiography (FA), and ICGA (in majority of cases; several cases were not diagnosed with ICGA due to dye shortage) (Visucam 524, 2019, Carl Zeiss Meditec AG, Jena, Germany). BCVA, FAF and OCTA were performed at the follow-up. SD-OCT measurements included the following parameters: central subfoveal thickness (CST), defined as the mean retinal thickness within the central 1 mm diameter circle; mean subfoveal thickness (MST), defined as the mean retinal thickness within the central 6 mm diameter circle; macular volume (MV), corresponding to the retinal volume within the central 6 mm circle; subfoveal choroidal thickness (SFCT), measured manually as the choroidal thickness beneath the foveola; and PED height, measured manually from Bruch’s membrane to the apex of the pigment epithelial detachment at the level of the retinal pigment epithelium (RPE). MNV was excluded on the basis of results of all performed examinations with special emphasis on OCTA. Complete resolution of PED was defined as the absence of any detectable elevation of the RPE above Bruch’s membrane or if the elevation was less than 10 μm. Partial resolution was defined as a persistent RPE elevation larger than 10 μm that was reduced in height compared with baseline measurements obtained from the Bruch’s membrane reference line.

Results considered in the analysis included measurements at presentation, at 1 month post PDT and 6 months post PDT. Besides the parameters of the affected eye, the analysis included also measurements of the fellow eye at baseline.

Duration and course of the disease before presentation were assessed on the basis of documents from the patient’s medical history and interview.

PDT procedure was conducted according to the half-dose protocol with a dose of verteporfin (3 mg/m^2^) and standard laser fluence 50 J/cm^2^ (Vitra 689, 2020, Quantel Medical, Cournon d’Auvergne Cedex, France). The amount of verteporfin required for each patient was calculated from their weight and height. Treatment planning was based on ICGA findings in conjunction with assessment of choroidal vascular morphology on SD-OCT. In all cases in which ICGA was performed, dilated and hyperpermeable choroidal vessels identified angiographically and corroborated on SD-OCT corresponded topographically to the location of the PED. Accordingly, the irradiation field using a 689 nm laser was defined to encompass the entire area of the PED with an additional safety margin of 500 μm. The rationale for this treatment area planning was based on the pathomechanism of CSC proposed by Chueung et al. [4], in which an isolated pigment epithelial detachment (PED) is considered a focal site of impaired retinal pigment epithelium (RPE) pump function secondary to localized choroidal vascular compromise. Accordingly, treatment was directed at areas of focal choroidal vascular dilation responsible for the RPE detachment.

Comparison of baseline and post-PDT parameters was conducted for central and paramacular groups according to PED location. Additionally, fellow eyes were also evaluated and compared to affected ones in cases with unilateral involvement.

### Statistical Analysis

Normality of distribution was assessed using the Shapiro–Wilk test. Non-parametric procedures were applied due to small group sizes and lack of normality. Friedman’s test was performed to assess the changes over time in quantitative traits from baseline and over two check-ups. General estimating equations (GEEs) with robust standard errors were fitted to test between-group differences in the models with repeated measures. The Mann–Whitney test was used to assess differences in numerical traits between the two study groups at baseline. Fisher’s exact test was performed to assess between-group differences for dichotomous variables. McNemar’s test was carried out to estimate changes in the frequency of the dichotomous variables between the check-ups. Spearman’s rank correlation coefficients were computed to assess relationships between the numerical variables. A level of *p* < 0.05 was deemed statistically significant.

The general estimating equations (GEEs), which were the main statistical procedure for assessing the changes over time and between-group differences, employed the said robust standard errors based on the intra-subject correlations.

All the procedures were performed using Statistica™, release 13.3 (TIBCO Software Inc., Palo Alto, CA, USA).

## 3. Results

The results of the study were divided according to location of PED: central versus paramacular. Only one PED lesion per eye was detected in each patient. There was a strong predominance of male subjects in both study groups. Patients with central location of PED presented with longer disease duration, worse visual acuity, and higher central retinal thickness, retinal volume and PED height.

Baseline characteristics of the two study cohorts are provided in Table 2.

A comparison of affected eyes versus fellow eyes in unilateral pachychoroid PED is presented in Table 3.

Affected eyes presented with significantly higher central retinal thickness and choroidal thickness. In 24 of 34 affected eyes, past symptoms of CSC, such as subretinal fluid or pigment abnormalities, were identified. Moreover, in 19 subjects with unilateral pachychoroid PED, past or present CSC was also detected in nine fellow eyes (one patient with loss of the fellow eye was not included).

**Figure 1:**
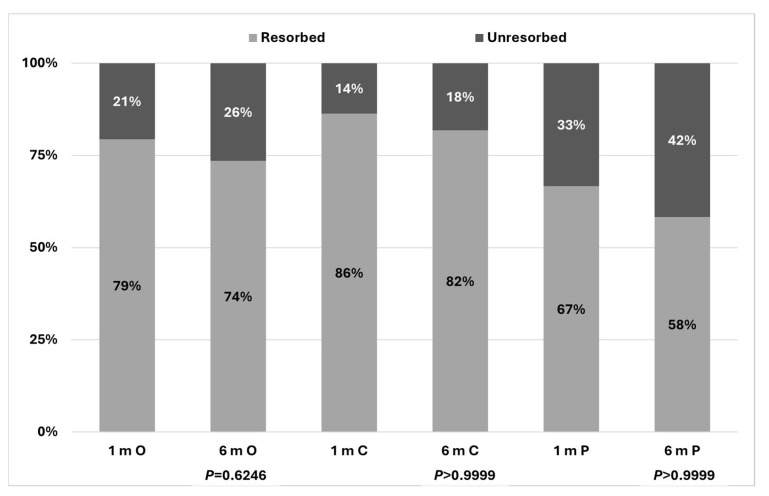
PED resorption in the study participants’ eyes 1 and 6 months after PDT by localization of PED (*p* = 0.0011). O—overall, C—central, P—paramacular, m—month; *p* values refer to the difference between the outcomes at month 1 and 6.

Results of total resorption of PED post half-dose PDT treatment are presented in Figure 1.

Overall resorption rate for the whole study cohort was 79.4% at 1 month, sustained at 73.5% at 6 months. For the central location of PED, these percentages were 86.3% and 81.8%, while for paramacular PED, they were 66.6% and 58.3%. During the first control examination at one month, no statistically significant difference between the two investigated localizations of PED was observed (*p* = 0.2113). During the second control assessment, no significant difference was noted, either (*p* = 0.2239).

**Figure 2:**
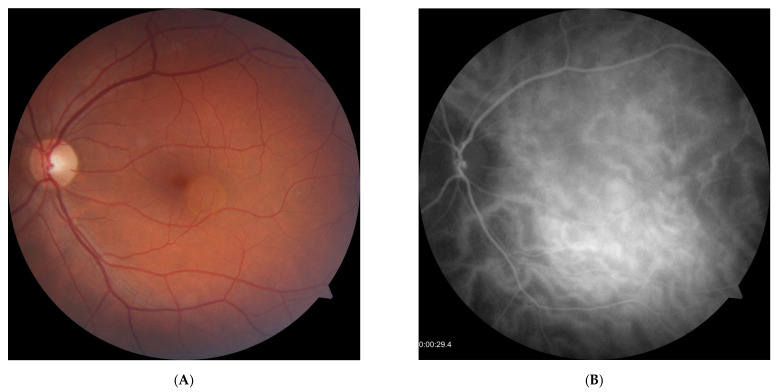

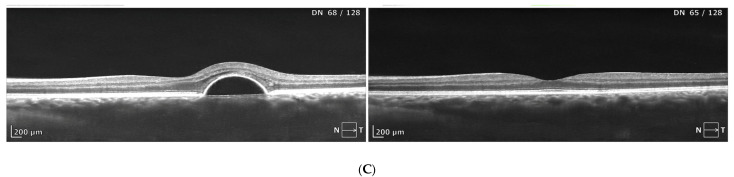
Complete resorption of central PED noted after one month post half-dose PDT (SD-OCT scan). (**A**)—color fundus photograph at baseline presents a round area of PED located in the macular center. (**B**)—ICGA photograph clearly identifies vessel dilation and leakage. (**C**)—SD-OCT scans before and after treatment show complete resorption of PED.

**Figure 3:**
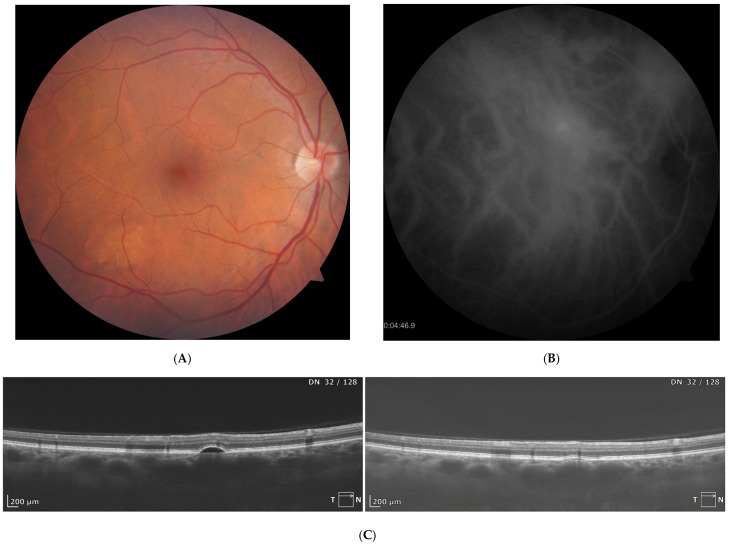
Complete resorption of paramacular PED noted at one month after half-dose PDT. (**A**)—color fundus photograph documents small roundish area of PED above the fovea. (**B**)—ICGA shows focal leakage in the location of PED. (**C**)—SD-OCT scans before and after hd-PDT present complete flattening of PED.

Examples of successful treatment of isolated PED with PDT are provided in Figure 2 (central location) and Figure 3 (paramacular location).

Mean BCVA improved significantly particularly in patients with central location of PED. Results of BCVA change after treatment for central and paramacular PED groups are presented in Table 4.

Results of morphological improvements measured with SD-OCT regarding CST, MST, MV, SFCT and PED height after PDT are presented in Table 5 and Supplementary Materials Tables S1–S3. Significant reductions in central retinal thickness, mean retinal thickness and retinal volume are noted in the subgroup with central location of PED. A significant decrease in mean SFCT was, however, not recorded.

Comparisons between eyes with positive and negative CSC history revealed significant differences in regard to final BCVA and SFCT. Patients with negative CSC history had better final visual acuity and lower SFCT compared to patients burdened with past CSC. There was also a tendency for smaller values of CST, MST and MV as well as higher PED resorption rate in the CSC-unrelated cohort. Nevertheless, there was no significant difference in PED resorption rate between the groups at 1 and 6 months (*p* = 0.6645 and *p* = 0.2250; Supplementary Materials Table S4), only a tendency for better results in eyes without CSC history (Table 6).

Nine patients with unresorbed PED at 6 months post PDT still showed significant reduction in mean PED height. Baseline PED height values decreased from mean 168 ± 123.61 μm to 136.13 ± 112.64 μm (*p* = 0.0477; Supplementary Materials Table S5). PED height post hd-PDT was not reduced in only three eyes compared to baseline values.

Comparisons between eyes with resorbed and unresorbed PED at 6 months following PDT demonstrated significant differences (Table 7). Eyes with an incomplete response were characterized by significantly lower baseline BCVA and more pronounced structural alterations, including greater PED height, increased CST, and the need for a larger PDT treatment spot.

A correlation analysis between best-corrected visual acuity (logMAR) at 6 months after PDT and baseline parameters was performed for the central PED subgroup. Spearman’s rank correlation coefficients (rho) revealed the following associations:

- BCVA and age: rho = 0.37, *p* = 0.0196
- BCVA and disease duration: rho = 0.49, *p* = 0.0017
- BCVA and PED height: rho = 0.22, *p* = 0.1753
- BCVA and CST: rho = −0.12, *p* = 0.4802
- BCVA and MST: rho = −0.35, *p* = 0.0314
- BCVA and MV: rho = −0.35, *p* = 0.0314
- BCVA and choroidal thickness: rho = 0.10, *p* = 0.5439
- BCVA and PDT spot diameter: rho = 0.54, *p* = 0.0003

Significant correlations were observed between final BCVA and age, disease duration, MST, MV, and PDT spot diameter. In particular, poorer final BCVA was associated with older age, longer disease duration, and larger PDT treatment area.

Treatment safety

No significant side effects were noted post PDT procedure, as evaluated by SD-OCT, FAF and CFP. FAF photographs performed at each follow-up visit demonstrated no increase in RPE loss compared to baseline evaluation. Subretinal neovascularization was not observed in any SD-OCT or OCTA scans.

## 4. Discussion

The results of our study demonstrate the efficacy of half-dose photodynamic therapy (hd-PDT) in achieving resolution or significant reduction in pachychoroid-related pigment epithelial PED. Beyond favorable morphological outcomes, patients experienced meaningful functional improvement, reflected by significant gains in BCVA. Notably, an increase in BCVA was observed in eyes with centrally located PED despite relatively high baseline visual acuity, underscoring the potential for functional enhancement even in cases with limited apparent visual reserve. Importantly, these therapeutic effects were achieved without the adverse events previously associated with PDT in pachychoroid spectrum diseases, such as RPE alterations or macular neovascularization [15,16,17].

Regarding anatomical response, a reduction in PED height without complete resolution was observed in 17.6% of cases (six eyes), whereas no response was documented in only 8.8% (three eyes). A slight attenuation of the therapeutic effect at 6 months was noted in a single case. Overall, these findings are consistent with previously reported outcomes of PDT in pachychoroid spectrum disorders, including CSC [18,19,20]. Repeated PDT may therefore be considered in eyes demonstrating a suboptimal response. Nevertheless, the potential for treatment failure should be acknowledged in a minority of patients with PPE treated with hd-PDT. Notably, suboptimal responders and non-responders exhibited features suggestive of more advanced disease at baseline, including lower BCVA and larger PED dimensions, which necessitated the use of a larger laser spot. Such cases appear inherently more challenging to manage and may carry a higher risk of incomplete therapeutic response.

Paramacular lesions demonstrated a tendency toward a less favorable response to hd-PDT compared with centrally located lesions. However, the difference in PED resorption rates did not reach statistical significance and may be attributable to the disparity in subgroup sample sizes. It should also be noted that the reported efficacy of hd-PDT in other non-central pachychoroid entities appears to be comparable. For instance, in peripapillary pachychoroid syndrome (PPS), the treatment efficacy has been reported at approximately 64% [21], which is somewhat lower than that observed in central CSC [18,19,20]. Nevertheless, the therapeutic effect of hd-PDT in paramacular lesions warrants further investigation in larger, adequately powered cohorts.

In the majority of cases analyzed in our cohort, isolated PEDs were associated with either a history of CSC or the presence of CSC in the fellow eye. It is plausible that, in certain instances, a solitary PED represented a residual manifestation of previously symptomatic CSC following resolution of subretinal fluid. However, a small subgroup of patients exhibited clinical features not directly attributable to CSC, as evaluated by FAF, FA, and SD-OCT. The only feature shared with CSC was increased choroidal thickness. Interestingly, these eyes were characterized by better final BCVA, lower choroidal thickness values, and a tendency toward higher PED resorption rates compared with eyes with a prior history of CSC symptoms. It may be hypothesized that, at an earlier stage of pachychoroid disease, photoreceptor damage is less advanced, which is subsequently reflected in more favorable functional outcomes. Furthermore, the RPE–choroid pump system in such cases may remain relatively preserved and capable of restoring homeostasis once choroidal vascular dilation is reduced by hd-PDT. This preserved functional integrity may also account for the more favorable anatomical response observed in this subgroup. In a larger study population, the differences in resorption rates between CSC-related and CSC-unrelated PED might have achieved statistical significance.

The identification of isolated PED in the absence of CSC history aligns with the conceptual framework proposed by Cheung et al. [4], who outlined a pathogenic mechanism underlying symptomatic CSC. According to this hypothesis, increased hydrostatic pressure within the choriocapillaris—resulting from choroidal congestion and impaired venous outflow (e.g., due to thick sclera, short axial length, or asymmetric vortex vein drainage)—leads to overload of the vortex venous system and triggers compensatory mechanisms in the RPE, including upregulation of RPE pump activity. Within this paradigm, isolated PEDs without subretinal fluid may represent a disease stage in which compensatory processes remain relatively effective and RPE integrity is largely preserved. Conversely, subretinal fluid associated with PED in CSC may regress spontaneously, leaving a residual PED as a sequela of prior symptomatic disease [22,23,24]. These scenarios likely represent distinct pathogenic pathways culminating in a similar morphological presentation. As previously reported, PPE frequently occurs in the fellow eyes of patients with exudative CSC, further supporting this interpretation.

Vascular choroidal remodeling and dysfunction of the RPE are currently regarded as central mechanisms in the development of symptomatic pachychoroid spectrum disorders [4,25]. Localized dilation of vessels within Sattler’s layer may exert mechanical compression on the choriocapillaris, leading to impaired perfusion and focal RPE decompensation [26,27]. This pathophysiological cascade may culminate in symptomatic CSC, but may also manifest as isolated PED in the absence of subretinal fluid [28].

Accordingly, detailed evaluation of choroidal vascularity should be considered a principal criterion for therapeutic decision-making, rather than reliance solely on increased choroidal thickness, which is known to vary substantially with age [29,30]. A similar rationale should be applied when assessing the effects of hd-PDT. Focal reduction in pathological choroidal vascular dilation appears to be of greater relevance to treatment efficacy than changes in overall choroidal thickness. Therefore, assessment of choroidal vascular remodeling with ICGA or optical coherence tomography—preferably swept-source OCT—should be incorporated both into treatment planning and into post-treatment evaluation of choroidal morphological response

The study population with pachychoroid-associated PED demonstrated a marked male predominance, with 85% male and 15% female patients. This distribution exceeds that typically reported in CSC, where the male-to-female ratio is approximately 3:1 [31,32,33]. However, our findings are consistent with prior reports focusing specifically on PPE, including Kishimoto et al., in which all participants were male, as well as Arif et al. [10] and Goto et al. [11], who reported male-to-female ratios of 16:3 and 13:2, respectively. These data suggest that this particular pachychoroid phenotype may exhibit an even stronger male predisposition than CSC.

To our knowledge, the present study represents one of the largest cohort of patients with isolated pachychoroid PED treated with PDT to date. Previous investigations addressing treatment of such cases have been limited to five studies [6,10,11,12,13] (Table 8)

It should be emphasized that these studies, besides the study by Hwang et al. (35 eyes), included relatively small sample sizes (ranging from 3 to 17 eyes) and employed heterogeneous PDT protocols. RPE alterations were reported following full-dose PDT in the study by Gupta et al., a complication not observed in our cohort treated with half-dose PDT [6].

The management of isolated PED must also be considered in light of its natural history, as observation without intervention may represent a reasonable approach in selected cases. The natural course of PPE has been evaluated in only two studies. Yagi et al. [34], in a large series of 148 eyes, examined fellow eyes of CSC patients with respect to the presence and progression of PPE. Over six years of follow-up, only 16.8% of PPE eyes developed an exudative phenotype. In the study by Arif et al. [10], 33% of untreated serous PEDs progressed to exudative CSC over a median follow-up of 11 months, whereas 38% demonstrated spontaneous PED resolution; however, this analysis was based on a relatively small sample of 22 eyes. Karakorlu et al. [35] reported transformation of 17.4% of PPE cases into symptomatic CSC without the development of subretinal neovascularization. More recently, Fong et al. documented neovascular transformation in only five of 108 eyes with PPE during one year of follow-up [36]. Collectively, these findings indicate that isolated PED may persist for extended periods without evolving into clinically manifest CSC.

In light of the visual improvement observed following hd-PDT in patients with pachychoroid-related PED—particularly in cases with central involvement—therapeutic intervention may be considered in selected symptomatic individuals. The decision to initiate invasive treatment should take into account the patient’s symptom burden as well as the stage and extent of disease. Our findings suggest that eyes presenting with lower baseline BCVA and larger lesions tend to demonstrate a less favorable response to hd-PDT compared with milder cases, indicating that baseline disease severity may influence treatment outcomes.

### Limitations of the Study

The primary limitations of this study are the relatively small sample size and the limited follow-up duration. It should be noted though that isolated PED is a rare condition seldom described as a distinct clinical phenomenon, which inherently restricts the ability to recruit a large cohort. Additionally, because patients traveled from various regions across the country to our center, maintaining a long-term longitudinal follow-up presented significant logistical challenges. A longer follow-up period is planned to further assess the durability and long-term sustainability of the therapeutic effect.

## 5. Conclusions

Half-dose photodynamic therapy was associated with favorable anatomical and functional outcomes in eyes with pachychoroid-related pigment epithelial detachment. The treatment led to complete PED resorption in the majority of cases and significant improvement in visual acuity, particularly in centrally located lesions. No clinically significant structural alterations or reductions in choroidal thickness were observed during follow-up. These findings suggest that hd-PDT may represent a therapeutic option for selected symptomatic patients with pachychoroid-related PED. However, given the retrospective design and limited follow-up duration, prospective controlled studies with longer observation and multimodal imaging correlation are warranted to further evaluate long-term efficacy and safety.

## Figures and Tables

**Table 1:** Baseline characteristics of the study cohort.

Parameter	*n* (%)
No. of participants	27 (n/a)
No. of eyes	34 (n/a)
Gender	
Female	4 (14.81)
Male	23 (85.19)
Localization of PED	
Central	22 (64.71)
Paramacular	12 (35.29)
Side	
Right eye	14 (41.18)
Left eye	20 (58.82)
	M (SD), Me (Q_1_–Q_3_)
Age [years]	45.57 (6.76), 44 (42–50)
Disease duration [months]	34.13 (32.28), 18 (12–48)
BCVA [logMAR]	0.22 (0.24), 0.15 (0.10–0.30)
Focal diameter of PDT spot [µm]	3685 (1131), 4000 (2500–4500)
CST [µm]	341.44 (137.35), 302 (272–359)
MST [µm]	322.79 (34.24), 318 (304–336)
MV [mm^3^]	9.13 (0.97), 8.98 (8.59–9.49)
SFCT [µm]	595.41 (73.83), 596 (546–650)
PED height [µm]	198.94 (125.55), 184 (157–184)

BCVA—best-corrected visual acuity, CST—central subfoveal retinal thickness, logMAR—logarithm of the minimum angle of resolution, M—mean, Me—median, MST—mean subfoveal retinal thickness, MV—macular volume, *n*—number, %—percentage, PDT—photodynamic therapy, PED—pigment epithelial detachment, Q—quartiles, SD—standard deviation, SFCT—subfoveal choroidal thickness.

**Table 2:** Baseline characteristics of the study participants’ eyes by localization of PED (*n* = 34).

	Central	Paramacular	*p* Value
	*n* (%)		
No. of eyes	22 (64.71)	12 (35.29)	n/a
Gender			
Female	3 (13.64)	1 (8.33)	>0.9999
Male	19 (86.36)	11 (91.67)	
	M (SD), Me (Q_1_–Q_3_)		
Age [years]	45.81 (6.56), 44 (42–48)	48.86 (7.36), 50 (44–52)	0.9037
Disease duration [months]	40.95 (37.00), 36 (12–48)	20.50 (12.52), 17 (12–24)	0.0135
BCVA [logMAR]	0.26 (0.26), 0.15 (0.10–0.30)	0.15 (0.17), 0.13 (0.00–0.25)	0.0483
PDT spot size [µm]	3513 (1067), 3750 (2500–4500)	4000 (1225), 4500 (3250–4500)	0.0820
CST [µm]	372.82 (158.48), 330 (287–379)	283.92 (55.42), 276 (264–311)	0.0007
MST [µm]	329.64 (39.43), 323 (307–338)	310.25 (16.86), 306 (300–321)	0.0105
MV [mm^3^]	9.32 (1.12), 9.11 (8.68–9.55)	8.77 (0.48), 8.65 (8.50–9.06)	0.0153
SFCT [µm]	601.54 (84.00), 601 (517–658)	584.17 (51.66), 582 (559–616)	0.3891
PED height [µm]	235.36 (131.38), 202 (148–310)	132.17 (82.58), 97 (76–199)	0.0005

BCVA—best-corrected visual acuity, CST—central subfoveal retinal thickness, logMAR—logarithm of the minimum angle of resolution, M—mean, Me—median, MST—mean subfoveal retinal thickness, MV—macular volume, *n*—number, %—percentage, PDT—photodynamic therapy, PED—pigment epithelial detachment, Q—quartiles, SD—standard deviation, SFCT—subfoveal choroidal thickness.

**Table 3:** Baseline characteristics in the study participants’ affected and fellow eyes (*n* = 19 eyes).

	**Affected Eye**	**Fellow Eye**	***p* Value**
	M (SD), Me (Q_1_–Q_3_)		
BCVA [logMAR]	0.20 (0.21), 0.15 (0.10–0.20)	0.31 (0.52), 0.10 (0.00–0.40)	0.5228
CST [µm]	368.10 (170.00), 311 (279–402)	309.37 (94.90), 287 (263–300)	0.0412
MST [µm]	332.74 (40.47), 323 (312–338)	335.79 (65.85), 311 (307–328)	0.2064
MV [mm^3^]	9.40 (1.15), 9.12 (8.81–9.56)	9.49 (1.86), 8.79 (8.68–9.26)	0.2101
SFCT [µm]	595.42 (80.34), 583 (559–650)	509.95 (54.85), 495 (469–531)	0.0002

BCVA—best-corrected visual acuity, CST—central subfoveal retinal thickness, logMAR—logarithm of the minimum angle of resolution, M—mean, Me—median, MST—mean subfoveal retinal thickness, MV—macular volume, Q—quartiles, SD—standard deviation, SFCT—subfoveal choroidal thickness.

**Table 4:** The observed dynamics of BCVA [logMAR] 1 and 6 months after PDT in the studied patients’ eyes by location of PED (*n* = 34).

Study Group and Phase	Statistical Parameter				*p* Value	
	M	SD	Me	Q_1_–Q_3_	Repeated	Between Group
Baseline O	0.22	0.24	0.15	0.10–0.30		
1 m O	0.13	0.18	0.10	0.00–0.15		
6 m O	0.10	0.16	0.05	0.00–0.10	<0.0001	
Baseline C	0.27	0.27	0.15	0.10–0.35		
1 m C	0.16	0.21	0.10	0.05–0.15		
6 m C	0.11	0.19	0.05	0.00–0.13	<0.0001	
Baseline P	0.15	0.17	0.13	0.00–0.25		
1 m P	0.09	0.13	0.05	0.00–0.10		
6 m P	0.07	0.11	0.03	0.00–0.08	0.0206	0.2097

M—mean; SD—standard deviation, Me—median, Q—quartiles, BCVA—best-corrected visual acuity, logMAR—logarithm of the minimum angle of resolution, PED—pigment epithelial detachment, PDT—photodynamic therapy, O—overall, C—central, P—paramacular.

**Table 5:** The observed dynamics of CST [µm] 1 and 6 months after PDT in the studied patients’ eyes by localization of PED (*n* = 34).

Study Group and Phase	Statistical Parameter				*p* Value	
	M	SD	Me	Q_1_–Q_3_	Repeated	Between Group
Baseline O	341.44	137.35	302	272–359		
1 m O	278.21	92.39	264	237–288		
6 m O	268.69	46.47	264	242–287	0.0001	
Baseline C	372.82	158.48	330	287–379		
1 m C	287.68	112.83	258	236–291		
6 m C	266.80	48.41	259	235–291	<0.0001	
Baseline P	283.92	55.42	276	264–31		
1 m P	260.83	28.14	271	246–278		
6 m P	271.83	44.97	269	256–279	0.6397	0.0116

M—mean; SD—standard deviation, Me—median, Q—quartiles, CST—central subfoveal retinal thickness, PED—pigment epithelial detachment, PDT—photodynamic therapy, O—overall, C—central, P—paramacular.

**Table 6:** The observed changes over time in BCVA [logMAR], CST [µm], MST [µm], MV [mm^3^], SFCT [µm], and PED height [µm] in the studied patients’ eyes 1 and 6 months after PDT by history of CSC (*n* = 34).

	Study Group and Phase	Statistical Parameter				*p* Value	
		M	SD	Me	Q_1_–Q_3_	Repeated	Between-Group
BCVA [logMAR]	Baseline N	0.11	0.06	0.10	0.10–0.15		
	1 m N	0.06	0.05	0.05	0.00–0.10		
	6 m N	0.03	0.05	0.025	0.00–0.05	<0.0001	
	Baseline P	0.27	0.26	0.20	0.10–0.40		
	1 m P	0.17	0.20	0.10	0.02–0.25		
	6 m P	0.13	0.18	0.05	0.00–0.18	<0.0001	0.0124
CST [µm]	Baseline N	324.60	58.08	311	279–359		
	1 m N	276.30	22.29	278	259–291		
	6 m N	282.10	26.02	282	263–293	<0.0001	
	Baseline P	348.46	157.98	302	271–360		
	1 m P	279.00	108.55	257	223–286		
	6 m P	300.46	135.84	260	228–287	0.0002	0.5960
MST [µm]	Baseline N	326.00	18.32	329	312–336		
	1 m N	318.30	11.99	321	311–329		
	6 m N	318.70	11.72	321	309–328	0.0001	
	Baseline P	321.46	38.79	314	303–331		
	1 m P	302.83	20.47	303	290–313		
	6 m P	304.00	27.16	304	286–311	<0.0001	0.0514
MV [mm3]	Baseline N	9.21	0.52	9.32	8.82–9.49		
	1 m N	9.00	0.34	9.08	8.80–9.30		
	6 m N	9.00	0.34	9.08	8.74–9.28	<0.0001	
	Baseline P	9.09	1.10	8.88	8.56–9.36		
	1 m P	8.56	0.58	8.57	8.22–8.85		
	6 m P	8.60	0.77	8.59	8.10–8.78	<0.0001	0.0567
SFCT [µm]	Baseline N	610.70	78.63	606	583–657		
	1 m N	634.60	68.28	645	638–664		
	6 m N	634.90	56.98	624	605–681	0.4493	
	Baseline P	589.04	70.80	574	542–626		
	1 m P	593.50	70.03	578	545–642		
	6 m P	573.79	63.63	575	515–620	0.1131	0.0055
PED height [µm]	Baseline N	182.10	95.78	175	103–221		
	1 m N	5.50	16.93	0	0–0		
	6 m N	18.60	40.89	0	0–0	<0.0001	
	Baseline P	205.96	135.12	184	88–279		
	1 m P	73.75	147.05	0	0–63		
	6 m P	69.54	144.73	0	0–77	<0.0001	0.1150

BCVA—best-corrected visual acuity, CST—central subfoveal retinal thickness, CSC—central serous chorioretinopathy, logMAR—logarithm of the minimum angle of resolution, M—mean, Me—median, MST—mean subfoveal retinal thickness, MV—macular volume, N—negative history of CSC, P—positive history of CSC, PED—pigment epithelial detachment, Q—quartiles, SD—standard deviation, SFCT—subfoveal choroidal thickness,

**Table 7:** Baseline characteristics of the study participants’ eyes according to PED resorption 6 months after PDT treatment (*n* = 34).

	Resorbed	Unresorbed	*p* Value
	*n* (%)		
No. of eyes	25 (73.52)	9 (26.47)	n/a
Gender			
Female	3 (16.67)	1 (11.11)	>0.9999
Male	15 (83.33)	8 (88.89)	
	**M (SD), Me (Q_1_–Q_3_)**		
Age [y]	45.20 (6.35), 44 (41–48)	47.00 (7.31), 47 (41–50)	0.4438
Disease duration [m]	30.09 (32.13), 18 (12–36)	36.56 (34.44), 18 (15–48)	0.3404
BCVA [logMAR]	0.15 (0.12), 0.10 (0.10–0.20)	0.41 (0.36), 0.20 (0.15–0.60)	0.0044
Focal diameter [µm]	3484 (1181), 3500 (2500–4500)	4244 (783), 4500 (4000–4500)	0.0117
CST [µm]	313.20 (103.00), 287 (270–326)	419.89 (191.25), 350 (330–402)	0.0001
MST [µm]	318.84 (20.59), 317 (304–333)	333.78 (58.14), 319 (302–339)	0.6642
MV [mm^3^]	9.01 (0.58), 8.95 (8.61–9.42)	9.45 (1.64), 9.09 (8.54–9.59)	0.6443
SFCT [µm]	600.52 (76.45), 604 (560–650)	581.22 (68.12), 568 (517–627)	0.4275
PED height [µm]	178.16 (124.45), 159 (87–215)	256.67 (116.03), 238 (163–314)	0.0053

*n*—number, %—percentage, M—mean, SD—standard deviation, Me—median, Q—quartiles; PDT—photodynamic therapy, PED—pigment epithelial detachment, BCVA—best-corrected visual acuity, logMAR—logarithm of the minimum angle of resolution, CST—central subfoveal retinal thickness, MST—mean subfoveal retinal thickness, MV—macular volume, SFCT—subfoveal choroidal thickness, m—months, y—years.

**Table 8:** Results of PDT treatment of isolated PED in former studies and present study.

Study (Year)	Material	PDT Protocol	PED Resolution (%) and Other Findings	Visual Acuity Outcomes
Gupta & Mohamed (2011) [6]	3 eyes of 3 patients	Standard PDT (full dose)	100% complete; RPE mottling in all cases post PDT	BCVA improved (2 cases) or stable (1 case)
Goto et al. (2012) [11]	15 eyes of 15 patients	Reduced-fluence PDT	93% (14 eyes) complete at 1–3 months; 100% overall improvement (reduction in PED height in 1 eye); significant reduction in subfoveal choroidal thickness; no complications	Significant improvement of mean BCVA from 0.08 to −0.01 logMAR at 3 months
Arif et al. (2018) [10]	9 eyes of 9 patients	Half-dose (1 eye)/standard dose (8 eyes) PDT	78% (7 out of 9 complete after 1 session); 1 eye complete after 3 sessions, 1 eye persisted despite treatment, no complications	Mean BCVA improved significantly from 0.8 to 1.0 Snellen
Hwang et al. (2019) [12]	35 eyes of 28 patients	Reduced-fluence PDT	80% complete resolution at 1 month sustained at the end of follow-up (mean 10.4 months)	Significant BCVA improvement from 0.15 to 0.09 logMAR at 3 months
Kishimoto-Kishi et al. (2025) [13]	17 eyes of 17 patients	Half-time PDT	64.7% (11 eyes) complete, 11.7% partial (2 eyes), 23.5% persisted unchanged (4 eyes) at 6 months post PDT; no complications	Significant mean BCVA improvement at 6 months from 0.05 to −0.02 logMAR, especially in foveal PED
Gawęcki et al. (2026) (present study)	34 eyes of 27 patients; 22 eyes central, 12 eyes paramacular PED	Half-dose PDT	Complete resolution in 79.4% at 1 month and 73.5% at 6 months	BCVA improvement significant from 0.22 to 0.1 logMAR at 6 months

PDT—photodynamic therapy; PED—pigment epithelial detachment BCVA—best-corrected visual acuity; RPE—retinal pigment epithelium; logMAR—logarithm of the minimum angle of resolution.

## Data Availability

The original contributions presented in the study are included in the article and Supplementary Materials. Further inquiries can be directed to the corresponding author.

## Supplementary Materials

The following supporting information can be downloaded at https://www.mdpi.com/article/10.3390/biomedicines14030620/s1. Table S1: The observed dynamics of MST [µm] 1 and 6 months after PDT in the studied patients’ eyes by localization of PED (*n* = 34); Table S2: The observed dynamics of MV [mm3] 1 and 6 months after PDT in the studied patients’ eyes by localization of PED (*n* = 34); Table S3: The observed dynamics of SFCT [µm] 1 and 6 months after PDT in the studied patients’ eyes by localization of PED (*n* = 34); Table S4: PED resorption in the study participants’ eyes 1 and 6 months after PDT by history of CSC (*n* = 34); Table S5: The observed changes in PED height [µm] 1 and 6 months after PDT in the studied patients’ eyes with unresorbed lesions (*n* = 9).

## Abbreviations

BCVA	Best-Corrected Visual Acuity
C	Central
CFP	Color Fundus Photography
CSC	Central Serous Chorioretinopathy
CST	Central Subfoveal Retinal Thickness
FA	Fluorescein Angiography
FAF	Fundus Autofluorescence
GEE	General Estimating Equations
hd-PDT	Half-Dose Photodynamic Therapy
ICGA	Indocyanine Green Angiography
logMAR	Logarithm of the Minimum Angle of Resolution
M	Mean
Me	Median
MNV	Macular Neovascularization
MST	Mean Subfoveal Retinal Thickness
MV	Macular Volume
*n*	Number
O	Overall
OCTA	Optical Coherence Tomography Angiography
P	Paramacular
PDT	Photodynamic Therapy
PED	Pigment Epithelial Detachment
PPE	Pachychoroid Pigment Epitheliopathy
PPS	Peripapillary Pachychoroid Syndrome
Q	Quartiles
RPE	Retinal Pigment Epithelium
SD	Standard Deviation
SD-OCT	Spectral-Domain Optical Coherence Tomography
SFCT	Subfoveal Choroidal Thickness
SRF	Subretinal Fluid
