# Supplementary material for: Pachychoroid-Related Pigment Epithelial Detachment Treated with Photodynamic Therapy
Source: Biomedicines. 2026 Mar 10;14(3):620. doi: 10.3390/biomedicines14030620 (PMC13024272; doi:10.3390/biomedicines14030620)
Supplement: Supplementary file 1 [file biomedicines-14-00620-s001.zip › biomedicines-4171356-supplementary.pdf]

Table S1. The observed dynamics of MST [μm] 1 and 6 months after PDT in the studied patients' eyes by localization of PED (n = 34)

Study group and phase	Statistical parameter				P value	
	**M**	**SD**	**Me**	**Q_1-Q_3**	**Repeated**	**Between group**
Baseline O	322.79	34.24	318	304-336		
1 m O	307.38	19.77	308	297-320		
6 m O	303.44	18.23	305	288-315	0.0004	
Baseline C	329.64	39.43	323	307-338		
1 m C	312.00	22.15	315	300-326		
6 m C	304.80	20.67	309	288-321	0.0029	
Baseline P	310.25	16.86	306	300-321		
1 m P	298.92	10.71	302	295-306		
6 m P	301.17	13.75	303	291-310	0.1073	0.1375

C – central, M – mean, Me – median, MST – mean subfoveal retinal thickness, O – overall, P – paramacular, PDT – photodynamic therapy, PED – pigment epithelial detachment, Q – quartiles, SD – standard deviation.

Table S2. The observed dynamics of MV [mm^3] 1 and 6 months after PDT in the studied patients' eyes by localization of PED (n = 34)

Study group and phase	Statistical parameter				P value	
	**M**	**SD**	**Me**	**Q_1-Q_3**	**Repeated**	**Between group**
Baseline O	9.13	0.97	8.98	8.59-9.49		
1 m O	8.69	0.56	8.70	8.40-9.05		
6 m O	8.59	0.52	8.63	8.18-8.91	0.0015	
Baseline C	9.32	1.12	9.11	8.68-9.55		
1 m C	8.82	0.62	8.91	8.48-9.21		
6 m C	8.65	0.59	8.74	8.18-9.11	0.0062	
Baseline P	8.77	0.48	8.65	8.50-9.06		
1 m P	8.45	0.30	8.54	8.36-8.66		
6 m P	8.51	0.39	8.56	8.25-8.76	0.1021	0.1252

C – central, M – mean, Me – median, MV – macular volume, O – overall, P – paramacular, PDT – photodynamic therapy, PED – pigment epithelial detachment, Q – quartiles, SD – standard deviation

Table S3. The observed dynamics of SFCT [μm] in 1 and 6 months after PDT in the studied patients' eyes by localization of PED (n = 34)

Study group and phase	Statistical parameter				P value	
	**M**	**SD**	**Me**	**Q_1-Q_3**	**Repeated**	**Between group**
Baseline O	595,41	73,83	596	546-650		
1 m O	605,59	72,08	597	548-657		
6 m O	595,66	68,52	586	555-639	0.5224	
Baseline C	601,55	84,00	601	517-658		
1 m C	610,73	73,92	624	548-657		
6 m C	605,05	78,72	606	557-664	0.7338	
Baseline P	584,17	51,66	582	559-616		
1 m P	596,17	70,73	581	549-660		
6 m P	580,00	45,80	581	555-620	0.5877	0.2388

C – central, CT – choroidal thickness, M – mean, Me – median, O – overall, P – paramacular, PDT – photodynamic therapy, PED – pigment epithelial detachment, Q – quartiles, SD – standard deviation.

Table S4. PED resorption in the study participants' eyes 1 and 6 months after PDT by history of CSC (n = 34)

**Follow-up**	**Negative history of CSC n/%**	**Positive history of CSC n/%**	**Overall**	**P value**
1 month	9 (90.00)	18 (75.00)	27 (79.41)	0.6445
6 months	9 (90.00)	16 (66.67)	25 (73.53)	0.2250
P value	0.4795	0.7728	0.7893	0.7385

CSC – central serous chorioretinopathy; n – number; % – percentage; PDT – photodynamic therapy.

Table S5. The observed changes of PED height [µm] 1 and 6 months after PDT in the studied patients' eyes with unresorbed lesions (n = 9)

Study phase	Statistical parameter				P value
	M	SD	Me	Q_1-Q_3	
**Baseline**	168,00	123,61	163	80-208	
**1 m**	122,78	125,20	63	55-158	
**6 m**	136,13	112,64	103	63-176	0.0477

M – mean; SD – standard deviation, Me – median, Q – quartiles, PED – pigment epithelial detachment, PDT – photodynamic therapy, P – paramacular
